# Clinical impact of serum soluble SLAMF7 in multiple myeloma

**DOI:** 10.18632/oncotarget.26196

**Published:** 2018-10-05

**Authors:** Mariko Ishibashi, Saori Soeda, Makoto Sasaki, Hiroshi Handa, Yoichi Imai, Norina Tanaka, Sakae Tanosaki, Shigeki Ito, Takeshi Odajima, Hiroki Sugimori, Toshio Asayama, Mika Sunakawa, Yuta Kaito, Ryosuke Kinoshita, Yasuko Kuribayashi, Asaka Onodera, Keiichi Moriya, Junji Tanaka, Yutaka Tsukune, Norio Komatsu, Koiti Inokuchi, Hideto Tamura

**Affiliations:** ^1^ Department of Hematology, Nippon Medical School, Tokyo, Japan; ^2^ Department of Microbiology and Immunology, Nippon Medical School, Tokyo, Japan; ^3^ Division of Hematology, Department of Internal Medicine, Juntendo University School of Medicine, Tokyo, Japan; ^4^ Department of Hematology, Gunma University, Gunma, Japan; ^5^ Department of Hematology and Oncology, IMSUT Hospital, The Institute of Medical Science, The University of Tokyo, Tokyo, Japan; ^6^ Department of Hematology, Tokyo Women's Medical University, Tokyo, Japan; ^7^ Department of Hematology, The Fraternity Memorial Hospital, Tokyo, Japan; ^8^ Department of Clinical Oncology, Iwate Medical University School of Medicine, Iwate, Japan; ^9^ Faculty of Health Science, Daito Bunka University Graduate School of Sports and Health Science, Tokyo, Japan; ^10^ Department of Preventive Medicine, Daito Bunka University Graduate School of Sports and Health Science, Saitama, Japan

**Keywords:** multiple myeloma, SLAMF7, soluble form, CS1, elotuzumab

## Abstract

The signaling lymphocytic activation molecule family (SLAMF7; also known as CS1 or CD319) is highly expressed on plasma cells from multiple myeloma (MM) as well as natural killer (NK) cells and is a well-known therapeutic target of elotuzumab. The objective of this study was to evaluate the clinical significance of serum soluble SLAMF7 (sSLAMF7) levels in patients with MM (n=103) and furthermore the impact of sSLMF7 on the antitumor activity of anti-SLAMF7 antibody. Thirty-one percent of MM patients, but not patients with monoclonal gammopathy of undetermined significance and healthy controls, had detectable levels of serum sSLAMF7, which were significantly increased in advanced MM patients. Further, MM in sSLAMF7-postive patients exhibited aggressive clinical characteristics with shorter progression-free survival times in comparison with sSLAMF7-negative patients. In responders to MM therapy, the levels of sSLAMF7 were undetectable or decreased compared with those before treatment. In addition, the anti-SLAMF7 antibody-mediated antibody-dependent cellular cytotoxicity of NK cells against MM cell lines was inhibited by recombinant SLAMF7 protein. Thus, our findings suggest that high concentrations of sSLAMF7, which could transiently suppress the therapeutic effects of elotuzumab, may be a useful indicator of disease progression in MM patients.

## INTRODUCTION

Multiple myeloma (MM), a malignancy of plasma cells, is characterized by the secretion of monoclonal immunoglobulin protein (also known as M protein) produced by the abnormal plasma cells [1, 2], accompanied by MM-related symptoms, i.e., hypercalcemia, renal insufficiency, anemia, and/or bone disease with lytic lesions [1]. Recent therapy for MM has changed drastically with the emergence of novel agents such as immunomodulatory drugs (lenalidomide and pomalidomide), proteasome inhibitors (bortezomib, carfilzomib, and ixazomib) and monoclonal antibodies (elotuzumab and daratumumab) [1–3]. In recent years, these novel agents have led to 3- to 4-fold prolongation of median survival times in MM patients [2, 3]. However, the resistance to antimyeloma agents in relapsed/refractory MM patients cannot be completely overcome and it is therefore necessary to understand the mechanism of resistance to targeted therapy in MM.

Several reports showed that soluble forms of various immune-associated molecules were detected in sera from patients with different types of cancer and that the levels reflected tumor progression or poor survival [4–8]. B cell maturation antigen (BCMA), which is highly expressed on MM cells, promotes the maintenance of cell proliferation and immunosuppression by the interaction with a proliferation-inducing ligand (APRIL) [9–12]. Its soluble form was elevated in serum from MM patients compared with that from healthy donors, and patients with high levels of soluble BCMA had shorter progression-free survival (PFS) and overall survival (OS) times than those with low levels [5, 13]. Similar to BCMA, the signaling lymphocytic activation molecule family 7 (SLAMF7; also known as CS1 or CD319) is highly expressed on plasma cells from MM patients and thus is the target of the humanized monoclonal antibody elotuzumab for MM treatment [14, 15]. SLAMF7 is also expressed on natural killer (NK) cells and increases NK cell activation by a mechanism dependent on an adaptor protein, Ewing's sarcoma-associated transcript 2 (EAT-2) [16, 17]. In contrast, SLAMF7 functions were not understood in MM cells lacking EAT-2, but SLAMF7 molecules on MM cells mediate cell adhesion to bone marrow (BM) stromal cells [18, 19]. The mechanisms of action of elotuzumab are: direct activation of NK cells via the EAT-2-mediated signaling pathway by binding with SLAMF7 on NK cells; induction of NK-mediated antibody-dependent cell-mediated cytotoxicity (ADCC); and interruption of the SLAMF7-mediated adhesion of MM cells to BM stromal cells [19, 20]. Treatment with elotuzumab in combination with lenalidomide and dexamethasone (Rd) in patients with relapsed/refractory MM improved PFS and OS in comparison with the control therapy of Rd alone [21, 22]. Tai and colleagues have recently identified soluble SLAMF7 (sSLAMF7) in serum from MM patients [18]. We investigated the concentration of serum sSLAMF7 and its clinical significance in MM. Furthermore, we examined whether sSLAMF7 could affect the antitumor activity of anti-SLAMF7 antibody.

## RESULTS

### Patients

The concentrations of sSLAMF7 were evaluated in serum from 103 patients with newly diagnosed MM (18 asymptomatic and 85 symptomatic MM); patient characteristics are shown in Table 1. According to International Scoring System (ISS) criteria, 24 (23.3%), 39 (37.9%), and 40 (28.8%) patients had stage I, II, and III diseases at diagnosis, respectively. According to Revised ISS (R-ISS) criteria, 16 (15.5%), 56 (54.4%), and 27 (26.2%) patients had stage I, II, and III diseases at diagnosis, respectively and 4 (3.9%) could not be classified. The median follow-up period from the time of diagnosis for all MM patients was 14 months (range 0–42 months).

**Table 1:** Patient characteristics

Characteristic (n=103)		No.
Age	median (range)	71 (32–88)
Sex	male/female	51/52
Diagnosis		
Asymptomatic MM		18
Symptomatic MM		85
Stage		
Internal Staging System (ISS)	l/ll/lll	24/39/40
Revised ISS	l/ll/lll/unknown	16/56/27/4
Durie-Salmon (DS) stage	l/ll/lll/unknown	14/24/61/4
DS type	A/B/unknown	81/18/4

### Detection of sSLAMF7 in serum of MM patients

**Figure 1:**
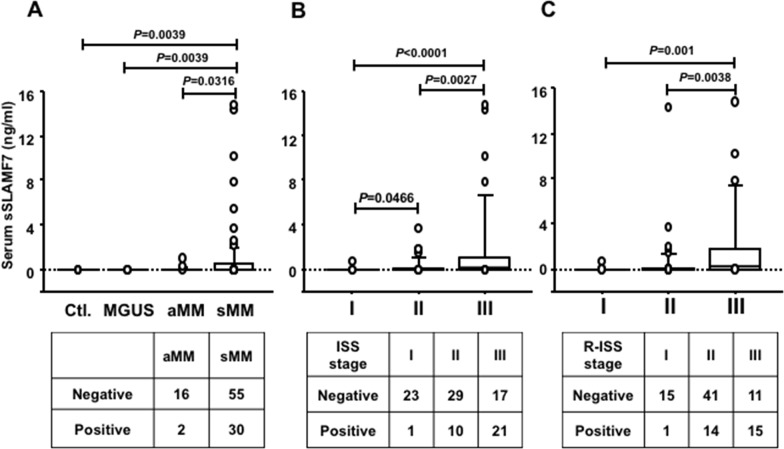
Circulating serum sSLAMF7 levels in MM patients according to disease stage **(A)** Comparison of serum sSLAMF7 levels among healthy controls (Ctl., n=16) and patients with MGUS (n=16), asymptomatic MM (aMM, n=18), and symptomatic MM (sMM, n=85). Comparison of serum sSLAMF7 levels in MM patients according to the ISS **(B)** and R-ISS **(C)** stage (Table 1). The lower tables in each graph indicate the number of MM patients positive and negative for serum sSLAMF7.

Thirty-one percent of MM patients (32 of 103), but no monoclonal gammopathy of undetermined significance (MGUS) patients (n=16) or controls (n=16), had detectable sSLAMF7 in serum (range: 0.091–14.7 ng/ml). sSLAMF7 levels in symptomatic MM patients were significantly higher than in asymptomatic ones (P=0.0316, Figure 1A). Furthermore, the levels of sSLAMF7 in MM patients with ISS and R-ISS stage III were markedly increased in comparison with those in patients with ISS and R-ISS stages I and II, respectively (Figure 1B and 1C). The association between sSLAMF7 detection and ISS or R-ISS stage was significant in 2×3 contingency table analysis (P<0.0001 and P=0.0006, respectively; Figure 1B and 1C). On the other hand, the percentage of plasma cells in BM cells was significantly higher in sSLAMF7-positive than in SLAMF7-negative patients, but was not correlated with sSLAMF7 concentrations (Supplementary Figure 1A and 1B). No association was found between sSLAMF7 concentrations and the SLAMF7 mRNA levels in CD138^+^ plasma cells (Supplementary Figure 1C and 1D).

### Clinical characteristics of sSLAMF7-positive MM patients

To determine whether sSLAMF7 may be a useful indicator of disease progression in MM, we compared the clinical characteristics between sSLAMF7-posotive (n=32) and -negative (no sSLAMF7 detected; n=71) patients. sSLAMF7-positive patients had higher ISS and R-ISS scores in comparison with the sSLAMF7-negative group (P=0.0002 and P=0.0013, respectively; Table 2).

**Table 2:** Comparison of clinical features between serum sSLAMF7-positive and -negative MM patients

		sSLAMF7		P value
		Negative (n=71)	Positive (n=32)	
Characteristic				
Age	median (range), years	71 (42–88)	70 (32–88)	NS
Gender	male/female	34/37	17/15	NS
Diagnosis	symptomatic/symptomatic	16/55	2/30	0.0518
M protein	IgG/IgA/IgM/BJP/unknown	47/12/0/8/4	21/3/0/7/1	NS
Ig type	κ/λ/unknown	34/29/8	18/13/1	NS
ISS	l/ll/lll	23/29/19	1/10/21	0.0002
R-ISS	l/ll/lll/unknown	15/42/12/2	1/14/15/2	0.0013
DS	l/ll/lll/unknown	12/18/38/3	2/6/23/1	NS
DS type	A/B/unknown	63/5/3	18/13/1	<0.0001
Bone lesions	0/1/2/3/unknown	24/7/6/29/4	6/4/6/14/2	NS
First-line therapy	Old doublet (MP/MD)	5	3	NS
	New doublet (BD/Rd)	29	20	
	Triplet (VMP/VCD/MPT/VRD)	22	8	
	Unknown	15	1	
Therapeutic response	≥VGPR/≤PR/Unknown	13/29/29	1/28/3	0.00514
Laboratory data				
Bone marrow plasma cells (%)		30.7 ± 17.3	42.7 ± 25.9	0.0025
White blood cell count (/μl)		5179 ± 2100	6257 ± 2597	0.0579
Hematocrit (%)		31.1 ± 6.40	28.0 ± 4.39	0.0268
Hemoglobin (g/dL)		10.4 ± 2.34	9.19 ± 1.43	0.0206
Platelets (×10^4^/μL)		17.9 ± 19.3	18.2 ± 9.0	NS
LDH (IU/L)		224 ± 68.7	303 ± 310	0.0213
Creatinine (mg/dL)		1.33 ± 0.93	3.07 ± 4.30	<0.0001
eGFR (mL/min)		45.6 ± 28.7	42.7 ± 30.1	0.0007
Corrected calcium (mg/dL)		9.73 ± 0.93	10.7 ± 1.84	0.0036
Albumin (g/dL)		3.12 ± 0.69	3.11 ± 0.80	0.0604
CRP (mg/dL)		0.33 ± 0.54	1.52 ± 2.66	0.0005
β2-microglobulin (μg/mL)		6.73 ± 4.96	14.2 ± 18.2	<0.0001
IL-6 (pg/mL)		6.57 ± 14.4	17.7 ± 21.6	0.0010
Cytogenetic abnormalities (+/−/unknown)				
t (4;14)		7/58/6	5/23/4	NS
t (14;16)		1/50/19	0/17/15	NS
del (17)		5/58/8	3/23/6	NS
del (13)		35/24/12	10/12/10	NS

Abbreviations: ISS, Internal Staging System; R-ISS, Revised ISS; DS, Durie-Salmon; MP, melphalan plus prednisone; MD, melphalan plus dexamethasone NS; BD, bortezomib plus dexamethasone; Rd; lenalidomide plus dexamethasone; VMP, bortezomib, melphalan, and prednisone; VCD, bortezomib, cyclophosphamide, and dexamethasone; MPT, melphalan, prednisolone and thalidomide; VRD, bortezomib, lenalidomide, and dexamethasone; VGPR, very good partial response; PR, partial response; LDH, lactate dehydrogenase; eGFR, estimated glomerular filtration rate; CRP, C-reactive protein; NS, not significant.

**Figure 2:**
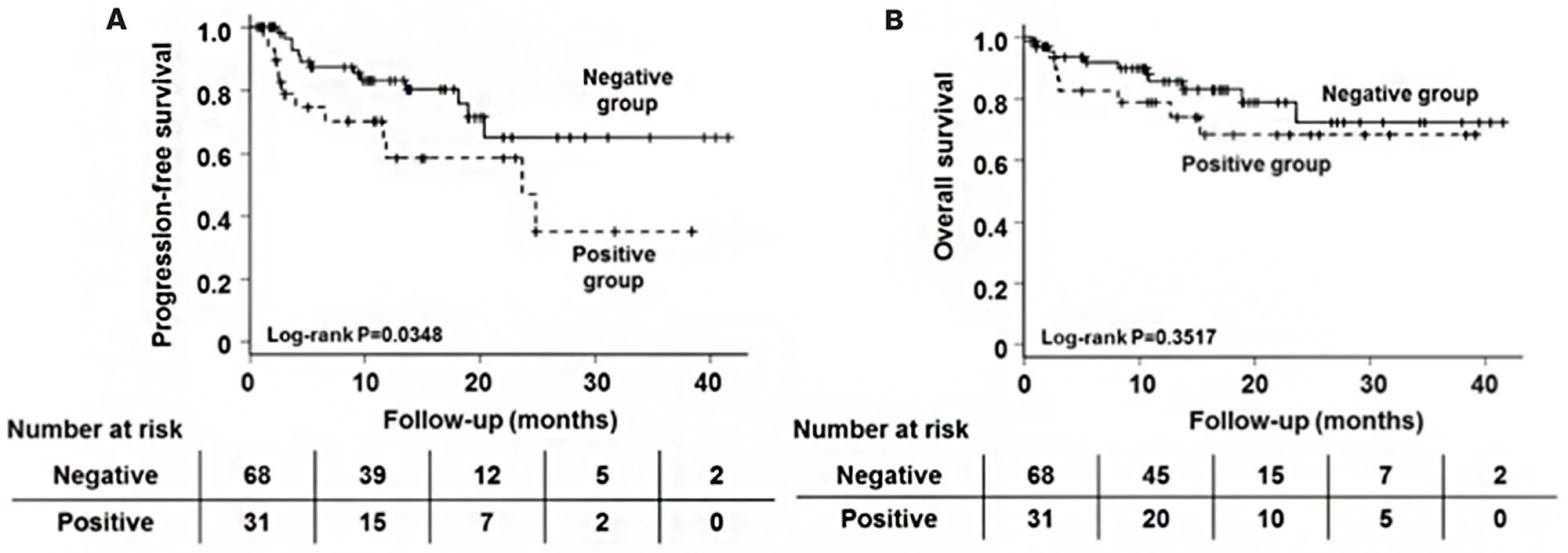
Kaplan–Meier estimates of PFS **(A)** and OS **(B)** in MM patients positive (n=68) and negative for sSLAMF7 (n=31).

There was no significant difference in PFS and OS between the patients treated with old doublets (melphalan and prednisolone [MP] or dexamethasone), new doublets (bortezomib/dexamethasone [BD] or lenalidomide/dexamethasone), and triplets (bortezomib/MP, BD/cyclophosphamide, MP/thalidomide, BD/lenalidomide) (data not shown). Table 2 shows that there was no difference in the distribution of those 3 regimens between sSLAMF7-positive and sSLAMF7-negative patients. Next, we assessed the association between the response after induction therapies and sSLAMF7-positivity. sSLAMF7-negative patients achieved significantly higher rates of very good partial response or better (P=0.00514, Table 2). Plasma cell percentages and serum levels of lactate dehydrogenase (LDH), creatinine, corrected calcium, C-reactive protein (CRP), β2-microglobulin, and interleukin (IL)-6 were significantly higher, while hematocrit, hemoglobin, and estimated glomerular filtration rate (eGFR) values were markedly lower in sSLAMF7-positive patients (Table 2). Furthermore, sSLAMF7-positive patients had shorter PFS, with a median of 24 months (P=0.035; hazard ratio [HR] 2.28; 95% confidence interval [CI] 1.4–5.02; Figure 2A and Supplementary Table 1). However, multivariate analysis of PFS showed that sSLAMF7 detection was not an independent prognostic factor (Supplementary Table 1).

### Serum sSLAMF7 concentrations after antimyeloma treatment in MM patients

**Figure 3:**
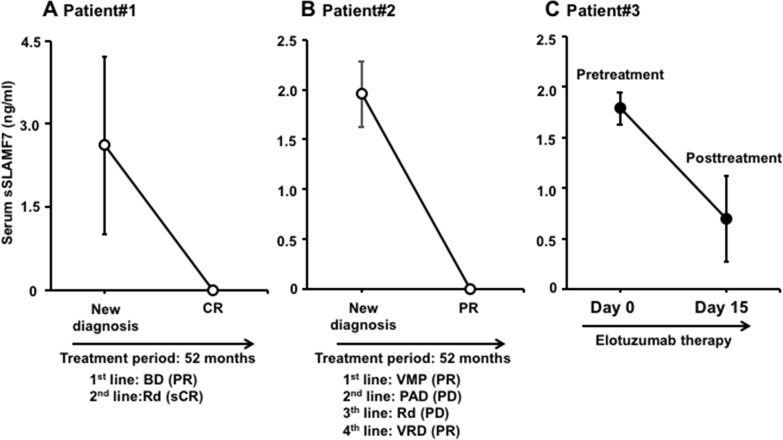
Levels of serum sSLAMF7 in MM patients before and after/during antimyeloma therapy **(A and B)** MM patients who received antimyeloma therapy and achieved a complete response (CR) or partial response (PR). **(C)** An MM patient was treated with elotuzumab weekly for 2 cycles. BD, bortezomib-dexamethasone; Rd, lenalidomide-dexamethasone; VMP, bortezomib-melphalan-prednisone; PAD, bortezomib-doxorubicin-dexamethasone; VRD, bortezomib-lenalidomide-dexamethasone; sCR, stringent complete response; PD, progressive disease.

Next, to investigate sSLAMF7 concentrations in patients after antimyeloma treatment, we focused on 2 SLAMF7-positive patients who achieved complement response (CR) or partial response (PR). MM patient #1 received BD as first-line therapy, and Rd as a second therapy, and thereafter achieved CR. MM patient #2 achieved PR after treatment with bortezomib and Rd as the initial therapy. The serum sSLAMF7 levels in those 2 patients became undetectable posttreatment (Figure 3A and 3B). Furthermore, refractory MM patient #3 was treated with the combination of elotuzumab and Rd, and sSLAMF7 levels were significantly decreased on day 15 of cycle 1 of compared with those pretreatment (Figure 3C). Therefore, levels of sSLAMF7 may decline with the response to antimyeloma therapy.

### Inhibition of anti-SLAMF7-mediated ADCC activity by recombinant SLAMF7 protein

**Figure 4:**
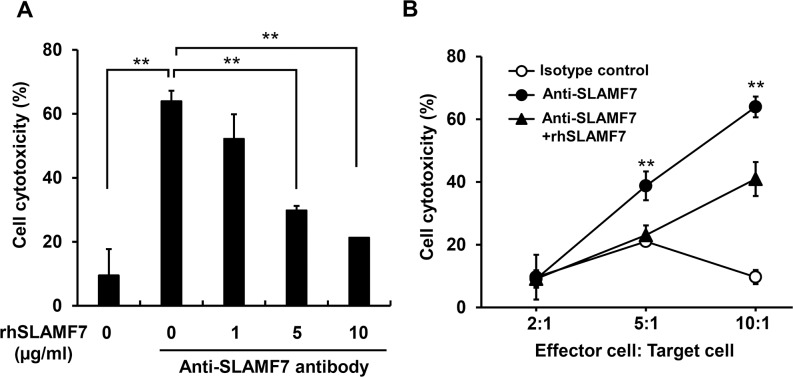
Inhibition of anti-SLAMF7-mediated ADCC activity against MM cells by rhSLAMF7 **(A)** Dose-dependent inhibition of anti-SLAMF7-mediated ADCC by rhSLAMF7. U266 cells treated with 20 μg/ml of anti-SLAMF7 antibody were co-cultured with NK-92MI cells at an effector : target ratio of 10:1. **(B)** In the ADCC assay, U266 cells treated with anti-SLAMF7 antibody were mixed with NK-92MI cells at different target : effector cell ratios. The data are expressed as mean ± SD of triplicate experiments. ^**^P<0.01.

We next examined whether sSLAMF7 impaired anti-SLAMF7 antibody-mediated ADCC activity. U266 cells treated with 20 μg/ml of mouse monoclonal anti-human SLAMF7 antibody and recombinant human SLAMF7 (rhSLAMF7) were co-cultured with NK-92MI cells for 4 h, and then ADCC activity by NK cells was determined using LDH-based cytotoxicity assays. The anti-human SLAMF7-mediated ADCC activity was suppressed by rhSLAMF7 in a concentration-dependent manner (Figure 4A). In addition, a high effector (NK cell): target (MM cell) ratio increased ADCC activity, which was inhibited by co-treatment with antibody and rhSLAMF7 (Figure 4B).

## DISCUSSION

The levels of serum soluble form of the cell-surface antigen BCMA [13, 23], programmed death-ligand 1 (PD-L1) [6], IL-6 receptor (IL-6R) [24], CD40 ligand (CD40L) [25], and vascular cell adhesion molecule (VCAM)-1 [26] were shown to be increased in MM patients and associated with MM disease progression. In the present study, we demonstrated that sSLAMF7 levels were significantly elevated in advanced-stage MM patients, and the presence of sSLAMF7 in MM patients was also correlated with advanced disease. In addition, we showed for the first time that MM patients with high sSLAMF7 levels had shorter PFS times.

Tai et al. also reported that sSLAMF7 was detected in 44% of MM patients, but not in MGUS patients and healthy controls, and was significantly higher in ISS stage II/III than in ISS stage I [18]. SLAMF7 molecules are highly expressed on normal and abnormal plasma cells from almost all MGUS and MM patients [19, 26]. High expression levels of SLAMF7 were also observed in MM patients including those with high-risk and low-risk molecular profiles and with and without cytogenetic abnormalities [14]. Although SLAMF7 gene expression in abnormal plasma cells was lower at relapse than at the time of diagnosis, its expression levels remained high at relapse [14]. Cell-surface expression levels of SLAMF7 were not associated with MM disease progression, while the presence of sSLAMF7 in serum from newly diagnosed MM patients reflected disease progression in our study. In our analysis, sSLAMF7-positive patients had significantly shorter PFS compared with sSLAMF7-negative patients in log-rank analysis, although sSLAMF7 detection was not an independent prognostic factor in multivariate analysis. The chi-squared test showed that significantly more sSLAMF7-positive patients were in R-ISS stage III (Figure 1C), and median PFS in those patients with R-ISS stage III and I/II disease was 200 and 757 days, respectively. Median PFS was not reached in sSLAMF7-negative patients in R-ISS stage III or stage I/II (data not shown). Those results suggest that the shorter time to achieve PFS in sSLAMF7-positive patients may be influenced by R-ISS stage III as a potential bias. In addition, no OS difference was found between sSLAMF7-postive and -negative patients in R-ISS stage III or I/II, suggesting that the presence of sSLAMF7 may not be associated with OS time. Furthermore, the median OS for both sSLAMF7-positive and -negative patients was not reached even after treatment with several novel drugs (Figure 2B). Many clinical trials demonstrated that new drug regimens improve the PFS but not OS time in refractory patients. Therefore, novel next-generation agents such as ixazomib, carfilzomib, plolidomide, elotuzumab, and daratumumab are expected to show excellent efficacy via different mechanisms, resulting in no difference between sSLAMF7-positive and sSLAMF7-negative patients with relapsed/refractory disease who are treated with those agents. Therefore, sSLAMF7 in serum from MM patients may be a useful indicator of disease progression.

Soluble BCMA levels in serum from untreated MM patients were markedly higher than those from MGUS patients and healthy controls and correlated with the proportion of BM plasma cells from MM patients [5]. In the present study, sSLAMF7 levels were not correlated with the percentages of BM plasma cells and mRNA levels of SLAMF7 in BM plasma cells from MM patients. However, sSLAMF7 was undetectable in sera from healthy controls with SLAMF7-expressing nonmalignant cells. Moreover, sSLAMF7 levels in serum from MM patients were undetected or decreased when achieving CR or PR after antimyeloma therapy. Thus, sSLAMF7 may be produced by abnormal plasma cells from MM patients by an unknown mechanism(s). Using immunoprecipitation techniques, Tai et al. demonstrated that sSLAMF7 in sera from sSLAMF7-positive MM patients was present in both its long and short forms, but was not detected in sSLAMF7-negative patient sera [18]. The long form is full-length SLAMF7, while the short form may represent a truncated version of the extracellular region [18]. However, the soluble form structure and the mechanism of production of sSLAMF7 remain unknown, and therefore further studies are needed to elucidate them.

SLAMF7 was reported to induce the proliferative activity of tumor cells in MM patients with the t(4;14) translocation [27]. sSLAMF7 is thought to bind SLAMF7 on myeloma cells, leading to tumor cell growth, and sSLAMF7-positivity was associated with R-ISS stage III, which may in turn be associated with a lower good response rate, as shown in Table 2. Further studies are needed to elucidate the function of sSLAMF7 in MM.

Elotuzumab is a SLAMF7-specific monoclonal antibody recognizing the C2-like domain within the extracellular domain [28]. In MM patients receiving combination therapy with elotuzumab, bortezomib, and low-dose dexamethasone, free sSLAMF7 levels that were not bound to elotuzumab in serum were significantly decreased when compared with baseline [29]. On the other hand, serum levels of total sSLAMF7, including those bound and not bound to elotuzumab, were markedly increased after elotuzumab treatment [29]. This suggests that serum sSLAMF7 binds to elotuzumab during treatment. In our study, rhSLAMF7 inhibited anti-SLAMF7-mediated ADCC activity on MM cells, indicating that a high concentration of sSLAMF7 in the BM microenvironment may suppress the effects of elotuzumab in MM patients. However, in most cases, elotuzumab doses administered for the treatment of MM are markedly higher than serum sSLAMF7 levels, and thus very high levels of elotuzumab not bound to sSLAMF7 may be present in the serum of patients during therapy.

In conclusion, our results suggest that the presence of serum sSLAMF7 may reflect MM disease progression. Although analyzing more samples with longer follow-up times could confirm these observations, our study indicates that serum sSLAMF7 could be a useful prognostic indicator in newly diagnosed MM.

## MATERIALS AND METHODS

### Patient samples

After obtaining informed consent, peripheral blood (PB) samples were obtained from individuals who underwent blood collection for diagnostic purposes at six clinical institutions from May 2012 to August 2015, and the patients were followed until April 2016. This study protocol and sampling were approved by the Institutional Review Board of each participating institution. The diagnoses were made according to International Myeloma Working Group criteria [30]. The individuals included 103 patients with newly diagnosed MM and 16 MGUS patients (Table 1). ISS, R-ISS, and Durie-Salmon staging were used to classify MM patients as in previous reports [31–33]. In some patients, PB samples were also obtained when their MM responded to standard chemotherapy or elotuzumab treatment. Healthy individuals were selected as controls (9 men and 7 women, with a median age of 55 [range 41–81] years).

### Cell lines

The human MM cell line U266 [34] was obtained from the American Type Culture Collection (ATCC; Manassas, VA, USA). The NK cell line NK-92MI (ATCC) was cultured in MEMα medium without nucleosides (Thermo Fisher, Waltham, MA, USA) containing 12.5% fetal bovine serum, 12.5% horse serum, 0.2 mM inositol, 0.1 mM 2-mercaptoethanol, 0.02 mM folic acid, 100 U/mL of penicillin, and 100 mg/mL of streptomycin.

### Detection of sSLAMF7 in sera from MM patients

sSLAMF7 concentrations were measured using the human SLAMF7 ELISA kit (MyBioSource, San Diego, CA, USA), according to the manufacturer's instructions. Each sample and standard protein were analyzed in duplicate; the minimum detectable level of sSLAMF7 was 78 pg/ml.

### Anti-SLAMF7-mediated ADCC assay

After pretreatment with 20 μg/ml of anti-SLAMF7 antibody (BioLegend, San Diego, CA, USA) at 4°C for 30 min, 1 × 10^4^ U266 cells per well were co-cultured with NK-92MI cells in round-bottomed 96-well plates at 37°C for 4 h. Cytotoxicity was determined using this cell culture supernatant in an LDH assay (Promega, Madison, WI, USA) according to the manufacturer's instructions. The percentage of cell killing was calculated as follow: % of lysis = [(experimental release – effector spontaneous release – target spontaneous release)/(target maximum – target spontaneous release)] × 100. rhSLAMF7 protein (ab151337) was purchased from Abcam (Cambridge, UK).

### Statistical analysis

Differences between two groups of data were determined using the χ^2^ or Fisher's exact test for categorical variables, and the Student *t* or Mann-Whitney U test for continuous variables. PFS was defined as the period from the date of new diagnosis to second-line treatment or to treatment cessation. OS was defined as the period from the date of new diagnosis until death or the last follow-up. PFS and OS curves were estimated using Kaplan–Meier analysis, and statistical differences between groups were compared in the log-rank test. Survival association with prognostic factors was determined by multivariate analysis using the Cox proportional hazards model. P values of less than 0.05 were considered to represent statistically significant differences. Statistical analyses were performed using SPSS version 23 software (Chicago, IL, USA).

## SUPPLEMENTARY MATERIALS TABLE AND FIGURE


