# Supplementary material for: Clinical impact of serum soluble SLAMF7 in multiple myeloma
Source: Oncotarget. 2018 Oct 5;9(78):34784–93. doi: 10.18632/oncotarget.26196 (PMC6205184; doi:10.18632/oncotarget.26196)
Supplement: Supplementary file 1 [file oncotarget-09-34784-s001.pdf]

Clinical impact of serum soluble SLAMF7 in multiple myeloma

SUPPLEMENTARY MATERIALS

Supplementary Table 1: Results of univariate and multivariate analyses

Variable		Univariate analysis			Multivariate analysis		
		HR	95% CI	P value	HR	95% CI	P value
Serum sSLAMF7 (Positive vs. Negative)		2.28	1.04-5.02	0.035	1.57	0.704-3.63	0.262
R-ISS	I vs. II	3.86	0.510-29.3	0.164	3.32	0.428-25.7	0.251
	I vs. III	9.42	1.17-75.8	0.021	7.72	0.926-64.4	0.059

HR, hazard ratio; 95% CI, 95% confidence interval.

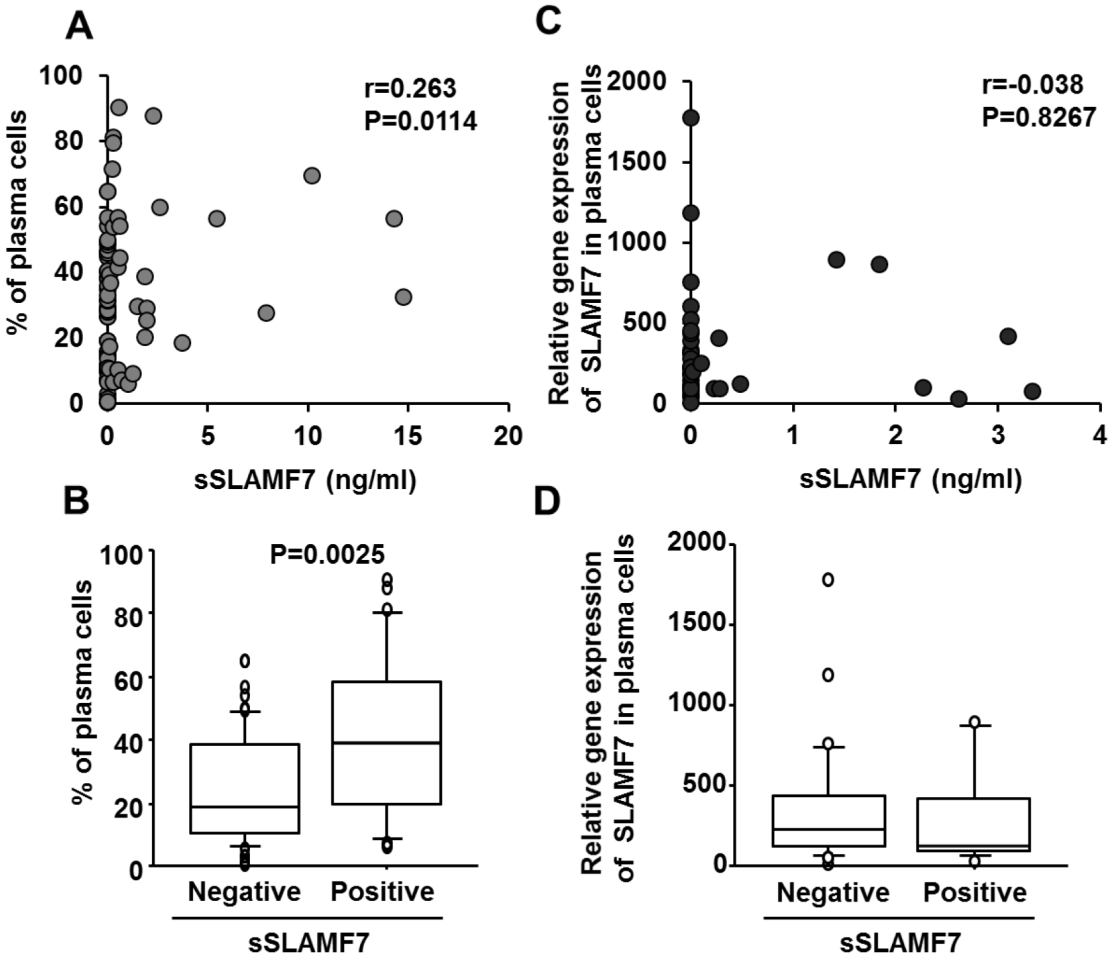

Supplementary Figure 1: Relationship between sSLMF7 and plasma cells from MM patients. **(A)** Correlation of serum sSLAMF7 with the percentage of bone marrow (BM) plasma cells from MM patients. **(B)** Comparison of the percentage of BM plasma cells between serum sSLAMF7-negative and -positive MM patients. **(C)** Correlation of serum sSLAMF7 with SLAMF7 mRNA levels in BM plasma cells. CD138^{+} plasma cells were isolated from BM mononuclear cells of MM patients using a CD138^{+} plasma cell isolation kit (Miltenyi Biotec, Bergisch Gladbach, Germany). After total RNA extraction, cDNA from total RNA was synthesized, and then quantification of mRNA using real-time PCR was performed. **(D)** Comparison of SLAMF7 mRNA levels of BM plasma cells between serum sSLAMF7-negative and -positive MM patients.
